# Ex vivo rabbit and human corneas as models for bacterial and fungal keratitis

**DOI:** 10.1007/s00417-016-3546-0

**Published:** 2016-11-14

**Authors:** Abigail Pinnock, Nagaveni Shivshetty, Sanhita Roy, Stephen Rimmer, Ian Douglas, Sheila MacNeil, Prashant Garg

**Affiliations:** 1 University of Sheffield, Sheffield, S10 2TA UK; 2 LV Prasad Eye Institute, Banjara Hills, Hyderabad, 500034 India; 3 University of Bradford, Bradford, BD7 1DP UK; 4 The Kroto Research Institute, North Campus, University of Sheffield, Broad Lane, Sheffield, S3 7HQ UK

**Keywords:** Ex vivo cornea, Microbial keratitis, Colony-forming units, Corneal model

## Abstract

**Purpose:**

In the study of microbial keratitis, in vivo animal models often require a large number of animals, and in vitro monolayer cell culture does not maintain the three-dimensional structure of the tissues or cell-to-cell communication of in vivo models. Here, we propose reproducible ex vivo models of single- and dual-infection keratitis as an alternative to in vivo and in vitro models.

**Methods:**

Excised rabbit and human corneoscleral rims maintained in organ culture were infected using 10^8^ cells of *Staphylococcus aureus*, *Pseudomonas aeruginosa*, *Candida albicans* or *Fusarium solani*. The infection was introduced by wounding with a scalpel and exposing corneas to the microbial suspension or by intrastromal injection. Post-inoculation, corneas were maintained for 24 and 48 h at 37 °C. After incubation, corneas were either homogenised to determine colony-forming units (CFU)/cornea or processed for histological examination using routine staining methods. Single- and mixed-species infections were compared.

**Results:**

We observed a significant increase in CFU after 48 h compared to 24 h with *S. aureus* and *P. aeruginosa*. However, no such increase was observed in corneas infected with *C. albicans* or *F. solani*. The injection method yielded an approximately two- to 100-fold increase (*p* < 0.05) in the majority of organisms from infected corneas. Histology of the scalpel-wounded and injection models indicated extensive infiltration of *P. aeruginosa* throughout the entire cornea, with less infiltration observed for *S. aureus*, *C. albicans* and *F. solani*. The models also supported dual infections.

**Conclusions:**

Both scalpel wounding and injection methods are suitable for inducing infection of ex vivo rabbit and human cornea models. These simple and reproducible models will be useful as an alternative to in vitro and in vivo models for investigating the detection and treatment of microbial keratitis, particularly when this might be due to two infective organisms.

## Introduction

Microbial keratitis is a major problem worldwide and is an important cause of vision loss and blindness. In vivo animal models, in vitro cell culture and ex vivo models have been used for investigating different aspects of this disease, including pathogenicity and treatment strategies [1–7].

In vivo studies require the use of a large number of animals to answer a research question. The welfare of these animals has become an important ethical issue [8], leading to the promotion of the philosophy of ‘replacement, reduction and refinement’ in the use of animals in research [9].

One way to overcome these issues is to use in vitro monolayer cultures of cells, including immortalised [5] or primary [4] corneal epithelial cells. However, these are not representative of the in vivo situation. They lack a three-dimensional (3D) structure and cross-talk between different epithelial cells, limbal cells and keratocytes. Consequently, advances have been made in 3D multi-layered tissue-engineered corneal constructs, and a few of these (EpiOcular™ from Mattek Corporation, Ashland, MA, USA, and HCE/corneal epithelium (SkinEthic) from Episkin, Lyon, France) are commercially available [10]. These have been used to study corneal pathogenesis [6, 11]. Whilst they possess the 3D architecture of their in vivo counterparts, these models often use immortalised cell lines and lack intrinsic innate immune molecules which occur in vivo.

Recently, there has been some interest in the use of ex vivo corneal models to study keratitis [12, 13]. Although these models lack immune elements, the 3D architecture remains, as do the intracellular innate immune molecules and cellular–stromal components. These models have been used for studying wound healing [14], microbial adherence [12] and molecular microbial pathogenicity [13]. In our laboratory, we have used ex-vivo corneal models to study corneal epithelial regeneration [15, 16] and to develop models of inflammation. We have also shown that by gently rocking media over the corneas, they can be maintained in culture for at least 4 weeks [17].

To the best of our knowledge, a comparison of bacterial and fungal infections and of mixed infections has not been undertaken in ex vivo corneal models. We report a comparison of single- and mixed-species infections in both rabbit and human corneas to better understand the use of these models in microbial keratitis.

## Materials and methods

### Materials

We used corneas from two types of rabbits—wild brown rabbits (Blackface Meat Company, Dumfries, Scotland) and New Zealand rabbits (University of Sheffield, from rabbits sacrificed at the end of a licenced study). There was no difference in the performance of corneas from these two types of rabbits. Cadaveric human corneas unsuitable for transplant were acquired from the Ramayamma International Eye Bank, L V Prasad Eye Institute, Hyderabad, India. All corneas were obtained following procedures approved by the institutional review board for the protection of human subjects.

Dispase II was obtained from Roche Diagnostics (Burgess Hill, UK), and Videne^®^ antiseptic solution was purchased from Ecolab (St. Paul, MN, USA). Mouse 3T3 fibroblasts (used in India) were from the American Type Culture Collection (ATCC; Manassas, VA, USA), and those used in the UK were an established J2 3T3 cell line originally from Professor Howard Green, USA. Epidermal growth factor was obtained from Invitrogen (Paisley, UK). For the culture of microorganisms, brain-heart infusion (BHI) agar and broth were purchased from Oxoid (Hampshire, UK) or HiMedia (Mumbai, India). All other reagents were obtained from Sigma-Aldrich (Dorset, UK) unless otherwise stated. Calcofluor-white was obtained from Sigma-Aldrich (Dorset, UK) and from HiMedia (Mumbai, India).

### Isolation of rabbit corneas

Corneas with sclera rims were dissected using a standard procedure including decontamination with povidone iodine, and were immediately placed into phosphate-buffered saline (PBS) [15].

### Ex vivo corneal organ culture

**Fig. 1:**
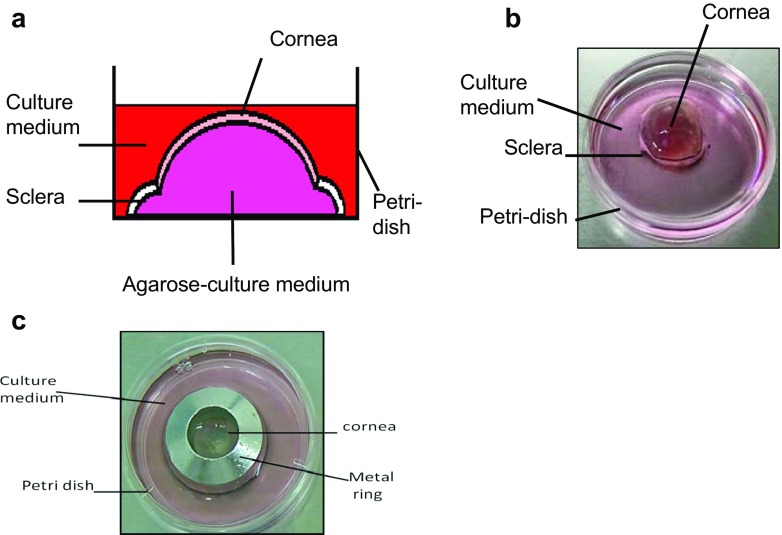
Schematic representation of a cross section (**a**) and a top-down image (**b**) of a corneoscleral button in organ culture. To infect corneas, a metal ring was placed on the corneoscleral button after wounding to form a seal, and bacteria/fungi were added to the surface of the cornea (**c**)

Organ cultures were as previously described [15, 18]. Rabbit and human corneoscleral buttons were placed epithelial side down in 35-mm petri dishes, and 500 μl Dulbecco’s modified eagle’s medium (DMEM)–agarose (0.5 % *w/v*) solution was pipetted into the endothelial side of the cornea. The solution was allowed to solidify, and the buttons were then inverted so that the epithelium was facing up (Fig. 1a and b). Culture medium (DMEM: Ham’s F12 [1:1] supplemented with 10 % fetal calf serum [FCS], 100 U ml^−1^ penicillin and 100 U ml^−1^ streptomycin, 2.5 μg ml^−1^ amphotericin B, 5 μg ml^−1^ insulin and 10 ng ml^−1^ epidermal growth factor [EGF]) was added to submerge the ex vivo corneas. Prior to infection, corneas were washed three times with PBS and incubated in antibiotic- and antifungal-free medium for at least 24 h to remove residual antimicrobials.

All experimental work was performed on rabbit corneas in the UK and on human corneas in India.

### Culture of bacteria and fungi

For rabbit corneas, laboratory strains of *S. aureus* (S-235), *P. aeruginosa* (SOM-1), *C. albicans* (SC5314) and *F. solani* strain (NCPF 2699), purchased from the National Collection of Pathogenic Fungi (UK), were used. For human corneas, ATCC cultures of *S. aureus* (25923), *P. aeruginosa* (27853) and *C. albicans* (90028) were used. All bacterial and fungal strains were cultured on brain-heart infusion (BHI) agar at 37 °C overnight and then maintained at 4 °C. For use in experiments, one colony was sub-cultured from agar into BHI broth and incubated overnight at 37 °C. Stationary-phase microbes were used in rabbit cornea experiments. For human corneal experiments, on the day of corneal inoculation, a fresh broth was inoculated, and exponential-phase bacteria/fungi were used based on predetermined growth curves.

### Infection of ex vivo corneas

Corneas were wounded with a scalpel (3 slashes vertically and 3 slashes horizontally), and a metal ring was placed on the corneoscleral button, creating a watertight seal. Into the centre of the ring, 10^8^ *S. aureus*, *P. aeruginosa*, *C. albicans* or *F. solani* were added (Fig. 1c), or the corneas were injected intrastromally (using a 26-gauge needle; Becton Dickinson, Oxford, UK) with the same number of organisms.

The infected corneas were incubated for 24 or 48 h at 37 °C, and were then homogenised and the resulting suspension serially diluted and spotted onto agar plates for colony enumeration. A set of infected corneas was also processed for histology and sections stained using Gram (bacteria) and periodic acid–Schiff (PAS) stains (fungi). Corneas not exposed to microbes were used as controls. Histological sections were imaged using a BX51 upright microscope and cell3D imaging software (Olympus, Essex, UK) in the UK or the ProgRes CapturePro 2.5 software (Jenoptik) in India.

### Imaging of microorganisms on the corneal surface

To visualise bacteria, 10^8^ *S. aureus* or *P. aeruginosa* were labelled using 1 mg ml^−1^ fluorescein isothiocyanate (FITC) for 1 h at 4 °C, followed by four washes with PBS. For fungi, whole corneas were covered with 1:1 calcofluor white in 10 % (*v/v*) potassium hydroxide for 10 min and washed three times with PBS. Bacteria- and fungi-infected corneas were imaged using fluorescence microscopy as described above.

### Statistical analysis

Box-and-whisker plots of colony-forming units (CFU) per cornea were plotted using GraphPad Prism 6 software. All comparisons were analysed using Student’s unpaired two-tailed *t* test, using Microsoft^®^ Excel (Microsoft^®^ Office, 2010). A p value ≤ 0.05 was considered significant.

## Results

### Macroscopic view of rabbit and human corneas

**Fig. 2:**
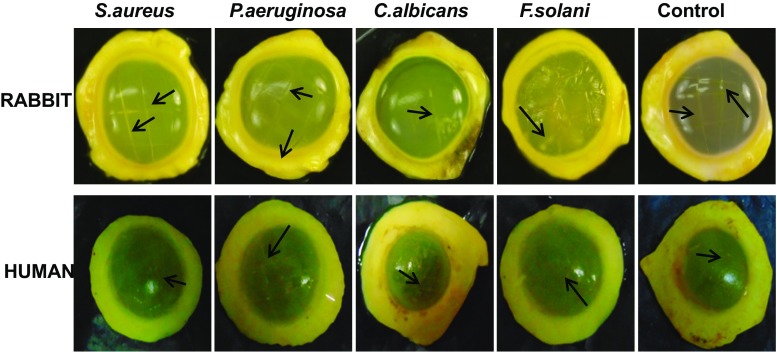
Fluorescein-stained rabbit and human corneas showing turbidity of infected versus non-infected corneas. Corneas were scalpel-wounded and exposed to *S. aureus*, *P. aeruginosa*, *C. albicans* or *F. solani* for 24 h. Corneas were briefly washed and stained with 0.5 mg ml^−1^ of fluorescein isothiocyanate for 30 min, washed again and photographed. *Arrows* indicate scalpel wounds

Corneas infected with bacteria and fungi showed a visible increase in haze compared with uninfected corneas (Fig. 2). The scratches were visible in all corneas, but were more evident in infected corneas than uninfected corneas (Fig. 2).

**Fig. 3:**
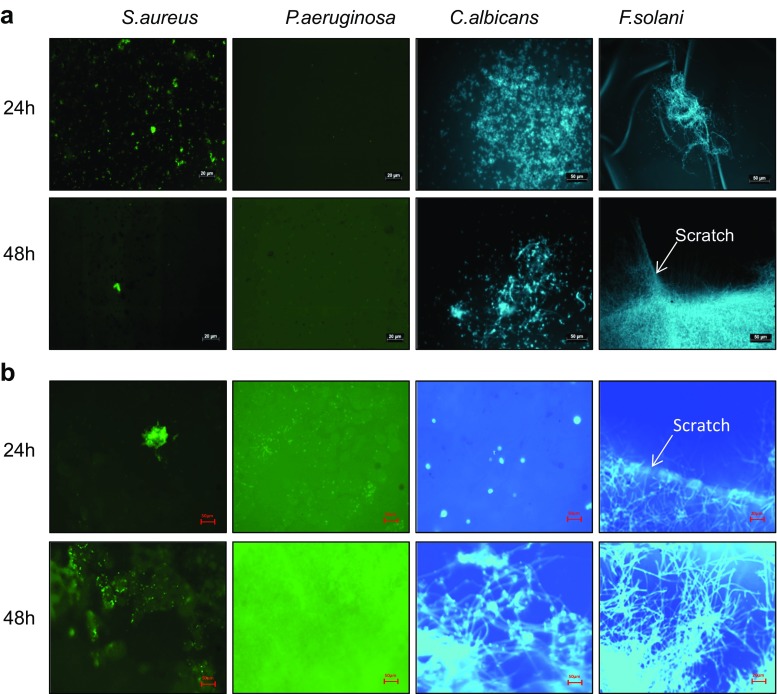
FITC-labelled *S. aureus* or *P. aeruginosa* were incubated with scalpel wounded rabbit (**a**) and human (**b**) corneas for 24 and 48 h, washed and imaged using a fluorescent microscope. Unlabelled *C. albicans* and *F. solani* were incubated with rabbit and human corneas for 24 and 48 h, washed, the model stained with Calcofluor White and imaged using a fluorescent microscope. The distribution of bacteria and fungi over the surface of the cornea and located within the scratch wound can be observed

FITC-labelled bacteria within infected rabbit and human corneas are shown in Fig. 3. It was observed that *S. aureus* cells covered the surface of the cornea at 24 h and 48 h, and at certain locations, clumps of bacteria ranging from 5 to 25 μm in diameter were detected. On the other hand *P. aeruginosa*-infected corneas showed fewer clumps than observed with *S. aureus*, after 24 and 48 h. A microscopic view of the surface of calcofluor white-stained *C. albicans*-infected corneas showed a more uniform spread of yeast and a few hyphal forms on the surface of the cornea after 24 h, which increased in both the distribution of individual yeast cells and the spread of hyphae by 48 h (Fig. 3). The distribution of *F. solani* at 24 h was more punctuated with hyphae at distinct places in both rabbit and human corneas (Fig. 3). After 48 h, the surface of the cornea was covered with a mat of fungi, where the hyphae could be observed extending into the scratch and in all directions, away from the fungal bulk (Fig. 3). The coverage of bacteria and fungi over the corneal surface was similar between rabbit and human corneas.

### Single-species infection of rabbit and human corneas

**Fig. 4:**
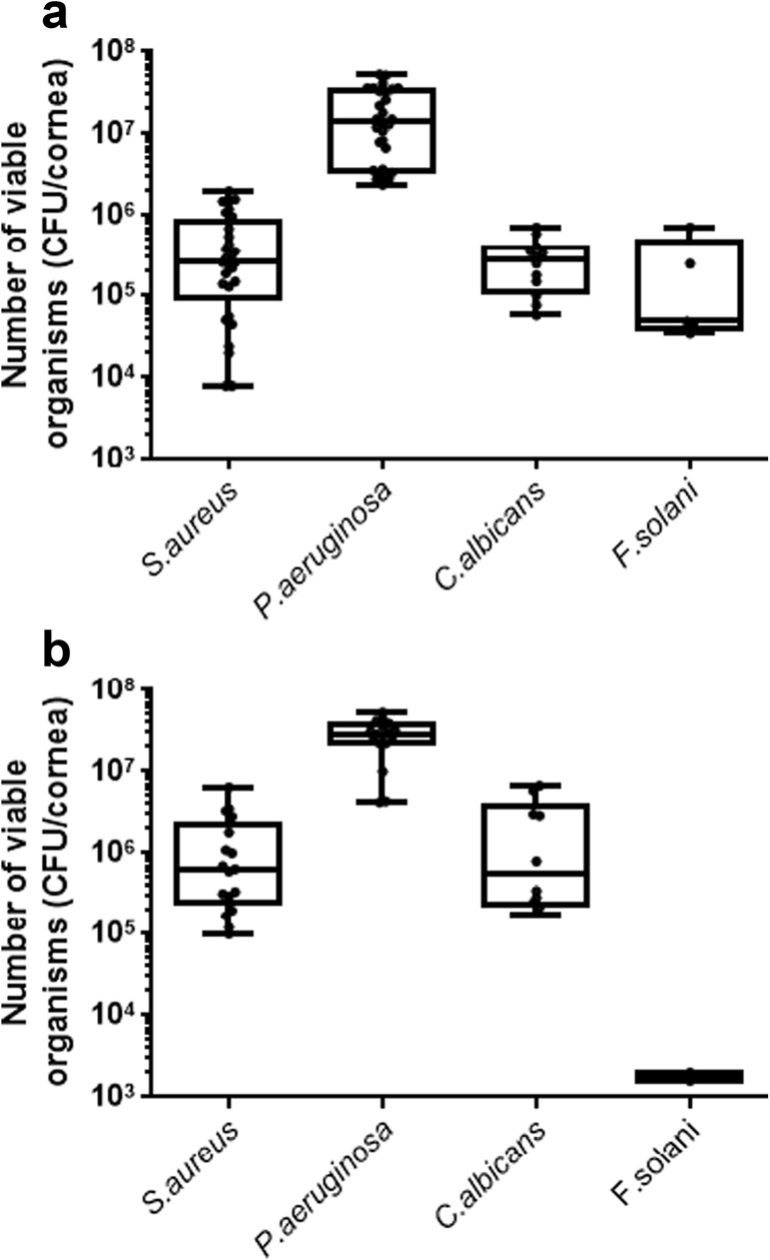
Single-species infection of ex vivo rabbit (**a**) and human (**b**) corneas. Ex vivo corneas were scratched six times with a scalpel and exposed to single-species inoculum of *S. aureus*, *P. aeruginosa*, *C. albicans* or *F. solani* for 24 h at 37 °C. The models were then homogenised, and the resulting suspension serially diluted and plated onto agar plates. The number of colony-forming units per cornea were plotted for each cornea. The box plot shows the minimum and maximum values depicted by the bars; the upper quartile, median and lower quartile are depicted by the top, middle and bottom horizontal lines, respectively

After 24 h, CFU recovered per cornea for *S. aureus*, *P. aeruginosa*, *C. albicans* and *F. solani* were as follows (Fig. 4): 5.1 ± 1.0 × 10^5^, 1.9 ± 0.3 × 10^7^, 3.0 ± 0.6 × 10^5^ and 2.5 ± 0.9 × 10^5^ CFU/rabbit cornea, respectively, and 3.8 ± 0.8 × 10^6^, 4.4 ± 0.6 × 10^8^, 1.9 ± 0.3 × 10^5^ and 1.8 ± 0.1 × 10^3^ CFU/human cornea, respectively. A significantly higher number of *S. aureus* and *P. aeruginosa* were recovered after 48 h of incubation in both rabbit (1.7 ± 0.3 × 10^6^ (*p* = 0.00005), 4.4 ± 0.7 × 10^7^ (*p* = 0.0009) and human corneas (1.5 ± 0.4 × 10^7^ (*p* = 0.0004), 6.5 ± 3.0 × 10^8^ (*p* = 0.0057), respectively, compared to yields at 24 h. There was no significant difference in the recovery of *C. albicans* or *F. solani* after 48 h, with 5.1 ± 1.5 × 10^5^ (*p* = 0.159) and 1.6 ± 0.7 × 10^6^ (*p* = 0.090) CFU/rabbit cornea, respectively, and 5.3 ± 1.6 × 10^5^(*p* = 0.108) and 2.1 ± 0.1 × 10^3^(*p* = 0.081) CFU/human cornea, respectively. In addition, there was approximately tenfold greater recovery of bacteria from human corneas than from rabbit corneas after both 24 and 48 h.

The injection method involved the introduction of bacteria and fungi into the stroma. Compared to the scalpel method, after 24 h, injection of a single-species organism resulted in higher CFU/cornea (*p* < 0.05) for all organisms, with the exception of *C. albicans* in human corneas, where no significant difference was observed (*p* = 0.057).

**Fig. 5:**
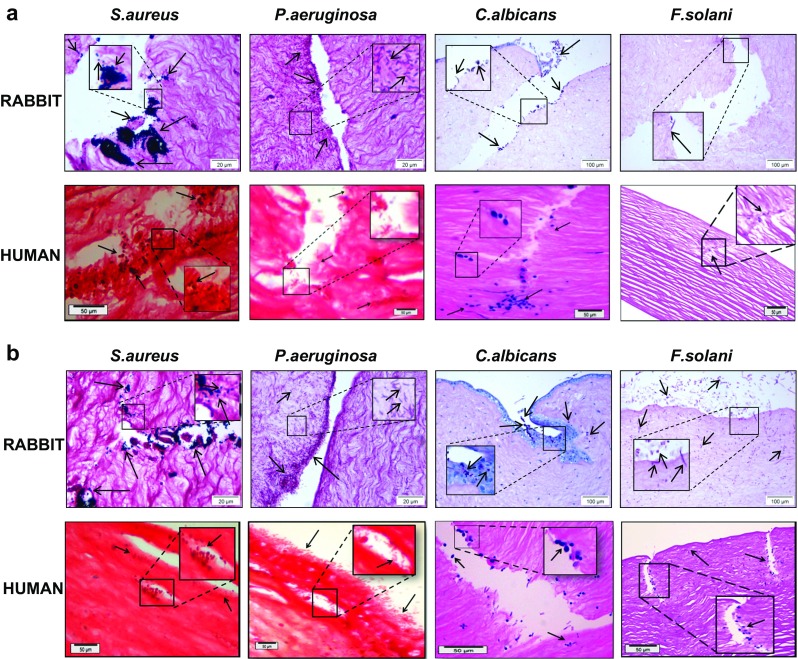
Histology of single-species infection of ex vivo rabbit and human corneas. Ex vivo rabbit and human corneas were scratched six times with a scalpel and exposed to a single-species inoculum of *S. aureus*, *P. aeruginosa*, *C. albicans* or *F. solani* for 24 h (**a**) or 48 h (**b**). Corneas were fixed in 10 % buffered formalin, embedded in paraffin, sectioned and stained using Gram stain (*S. aureus* and *P. aeruginosa*) or PAS stain (*C. albicans* and *F. solani*). Gram-positive (*purple*) cocci (*S. aureus*), Gram-negative (*pink*) rods (*P. aeruginosa*), and purple round yeast and hyphae (*C. albicans* and *F. solani*) can be observed at the epithelial surface and within the scratch, and are present in the stroma

The histology of single-species-infected corneas after 24 h is shown in Fig. 5a. Here, vast infiltration of *P. aeruginosa* can be seen covering the epithelium and entire stroma and infiltrating the Descemet’s membrane. This was independent of the method of inoculation (data not shown). The histology of the *S. aureus*-infected corneas was characterised by the concentration of the majority of organisms within the scratches (Fig. 5a). The number of bacteria within tissue sections correlated with the CFU/cornea data (Fig. 4).

The distribution of *C. albicans* within the corneal tissue was similar between the human and rabbit corneas. Yeast cells and hyphal elements were observed close to the scratch site, with no infiltration beyond 150 μm into the stroma (Fig. 5a). The number of hyphae and the infiltration of *C. albicans* cells into the stroma after 48 h did not differ from that observed after 24 h.

The tissue penetration by *F. solani* after scalpel wounding was less than that for *C. albicans* at 24 h, with infiltration not more than 10 μm into the stroma from the site of inoculation (Fig. 5a), which increased after 48 h in both number and depth of penetration.

### Two-species infection of corneas

**Fig. 6:**
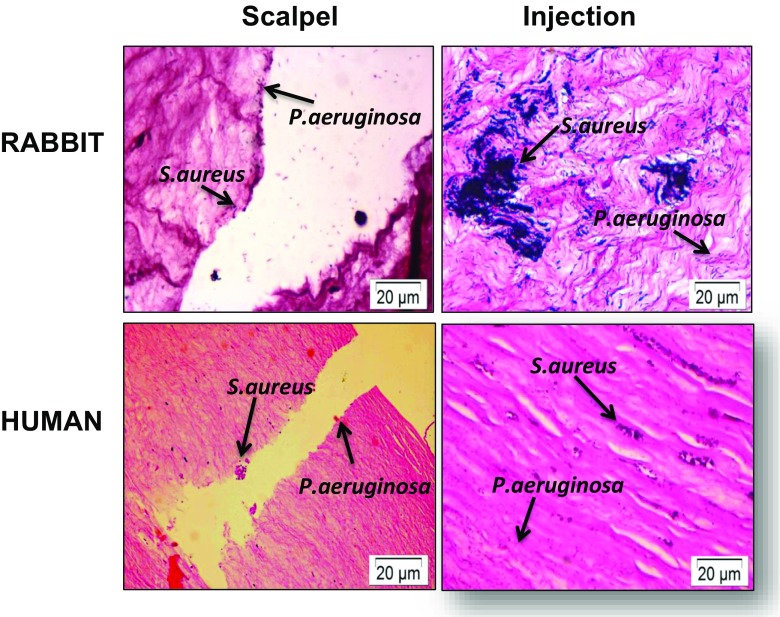
Histology of rabbit and human ex vivo models showing a mixed *S. aureus* and *P. aeruginosa* infection. At different sites within the same cornea, ex vivo corneas were intrastromally injected with 10^8^ *S. aureus* and 10^8^ *P. aeruginosa* and incubated for 24 h (injection). Alternatively, corneas were wounded with a scalpel, and 10^8^ *S. aureus* and 10^8^ *P. aeruginosa* were added to the surface of the cornea for 24 h (scalpel). Sections were Gram-stained and imaged to visualise *S. aureus* and *P. aeruginosa* within their injection sites at distinct locations within the stroma. *P. aeruginosa* shows widespread infiltration into the tissue, whereas *S. aureus* shows less infiltration

In the two-species infection model, we were able to recover both bacterial species from infected rabbit and human corneas (Table 1). Histological examination confirmed the quantitative colony count data (Fig. 6). As with the single-species model, infiltration of *P. aeruginosa* cells was found throughout the stroma, including Descemet’s membrane, whereas *S. aureus* showed little spread beyond the injection site.

**Table 1:** Numbers of CFU/cornea recovered from multi-bacterial/fungal infections after 24 h

A				
	RABBIT		HUMAN	
*S. aureus* - *P. aeruginosa*	*S. aureus*	*P. aeruginosa*	*S. aureus*	*P. aeruginosa*
	1.88 ± 0.6 × 10^6^	3.09 ± 0.9 × 10^7^	5.25 ± 1.6 × 10^5^	2.43 ± 3.2 × 10^8^
*C. albicans* - *P. aeruginosa*	*C. albicans*	*P. aeruginosa*	*C. albicans*	*P. aeruginosa*
	4.20 ± 1.6 × 10^6^	2.12 ± 0.9 × 10^8^	5.15 ± 6.6 × 10^5^	1.00 ± 1.2 × 10^8^
B				
	RABBIT		HUMAN	
*S. aureus* - *P. aeruginosa*	*S. aureus*	*P. aeruginosa*	*S. aureus*	*P. aeruginosa*
	9.26 ± 4.2 × 10^6^	5.83 ± 2.4 × 10^8^	2.50 ± 5.7 × 10^4^	3.72 ± 1.08 × 10^8^
*C. albicans* - *P. aeruginosa*	*C. albicans*	*P. aeruginosa*	*C. albicans*	*P. aeruginosa*
	4.25 ± 1.1 × 10^6^	6.84 ± 0.6 × 10^8^	4.05 ± 9.6 × 10^5^	7.65 ± 1.0 × 10^8^

Two multi-pathogen infections were investigated. These were a mixed *S. aureus*/*P. aeruginosa* and a mixed *C. albicans*/*P. aeruginosa* infection. A: EX vivo rabbit and human corneas were wounded with a scalpel and exposed to a mixture of 10^8^ of both organisms for 24 h B: Ex vivo rabbit and human corneas were intrastromally injected with 10^8^ cells of the first organism at 3–5 distinct locations, and then 10^8^ cells of the second organism were similarly injected at different sites. The corneas were incubated for 24 h, washed, homogenised, serially diluted and plated onto agar plates, and the CFU/cornea calculated. Data is expressed in CFU/cornea ± SEM of at least three independent experiments performed in triplicate

A mixed infection involving *C. albicans* and *P. aeruginosa* is the most commonly observed clinically [19]. In ex vivo models of this mixed infection, both organisms were recovered after 24 h following scalpel wounding, with *P. aeruginosa* showing dominance within the tissue by both colony counting (4.20 ± 1.6 × 10^6^ and 2.12 ± 0.9 × 10^8^ CFU/rabbit cornea and 5.15 ± 6.6 × 10^5^ and 1.00 ± 1.2 × 10^8^ CFU/human cornea for *C. albicans* and *P. aeruginosa*, respectively) and histology (Table 1 and Fig. 6). The level of recovery of both organisms was approximately the same regardless of the method of introduction of organisms.

## Discussion

Ex vivo models have previously been used to study corneal–microbial interactions [12, 13]. However, there have been no direct comparisons of single and mixed bacterial and fungal infections or a comparison of the infection of rabbit and human corneas. Here, we describe the numbers of viable organisms recovered from rabbit and human corneas after 24 and 48 h, showing histological images from scalpel wounding and intrastromal injection as ways of introducing organisms to the cornea.

A variety of methods have been described in the literature for introducing bacteria or fungi to experimental (in vivo, in vitro or ex vivo) corneas. These include the use of bacterial/fungal-inoculated contact lenses [20, 21], blotting paper and ethylene glycol tetraacetic acid (EGTA) [7], and mechanical removal of the epithelial surface [22, 23]. However, the most commonly described methods are corneal scratch [24, 25] and intrastromal injection [26, 27]. Therefore, we chose to scratch the corneas with a scalpel six times, so that the scratch revealed the upper stromal compartment, and also to introduce organisms intrastromally using an injection method. Although the injection method gave a greater yield of organisms than the scalpel method, we observed that the scalpel method mimicked clinical infection in which infection is initiated from an abrasion on the corneal surface [28]. Because it was thought that the infiltrative capacity of *P. aeruginosa* within the tissue might inhibit or prevent the growth and propagation of *S. aureus* and/or *C. albicans* when introduced together through a scalpel wound, we compared this method with intrastromal injection, injecting organisms at separate sites and thus preventing their interaction. However, the scalpel method did not prevent the recovery of *S. aureus* or *C. albicans*, suggesting that either method is suitable for establishing a mixed infection model. Therefore, we show that an infection can be induced in the ex vivo corneas for both single and multiple species using either scalpel wounding or intrastromal injection.

The following aspects of this model need further discussion:

1. A large bacterial/fungal inoculum was used, because this corneal model does not have a blood supply or immune system. Consequently, the damage that the inflammation causes to the local tissue, and which provides additional nutrients via the vasculature for the bacteria/fungi, was not present.
2. *S. aureus* was not typically found at the epithelial surface, but rather within the scratches, commonly in clusters, and not migrating into surrounding and deeper cornea. This has been described previously as well [29, 30]. The observed attachment of *S. aureus* at the stromal surface is also supported by the observations of Rhem et al. [29], who demonstrated that collagen-binding clinical *S. aureus* isolates expressing the *cna* collagen-binding gene showed enhanced tissue disruption compared to a *cna* ^−^ isogenic mutant. The cna protein is considered to be a virulence factor mediating bacterial adherence to the epithelial surface and the stroma, and neutrophil recruitment to the infection site. Of the two strains of *S. aureus* that we used in this study, the ATCC 25923 strain is known to express this gene [31], which could be the reason for the higher level of binding within the scratch than at the surface (it is unknown whether this gene is expressed in the local clinical strain, S235). According to reports in the literature, in vivo corneas infected with *S. aureus* by intrastromal injection returned bacterial counts of approximately 10^4^–10^7^ CFU/cornea [32, 33], depending on the number of bacteria in the starting inoculum and the length of time the bacteria were incubated with the eye. These values are in line with the recovery we obtained from ex vivo models, suggesting that our model is representative of an in vivo infection in terms of the number of bacteria recovered.
3. We did not observe any ulceration or corneal edema. This is because ex vivo corneas lack inflammatory cells that are primarily responsible for epithelial ulceration [32], stromal polymorphonuclear neutrophil (PMN) infiltration [34–36], and ulcer formation [37] seen in clinical *S. aureus* infection.
4. Compared to the *S. aureus* model, *P. aeruginosa*-infected corneas yielded a greater number of CFU/cornea and were seen infiltrating the entire cornea, despite having the same inoculum. This high level of infiltration has been shown to be the result of proteolytic bacterial enzymes, including type III secretion system-associated cytotoxins, exoenzyme U and exoenzyme S [38, 39], alkaline protease and elastase [40], which have been shown to contribute to corneal erosion [41]. In addition, host proteolytic enzymes also contribute to corneal ulceration [26]. We observed a softening of *P. aeruginosa*-infected corneas and increased opacity compared with control corneas, but no ulceration. As mentioned previously, this was due to the lack of an immune cell component in these ex vivo cultures.
5. Previous studies have reported an increase in the recovery of *P. aeruginosa* from corneas compared with the initial inoculum [42, 43]. However, we did not find this increased recovery of *P. aeruginosa*. Although we have no definitive explanation for this observation, one possible explanation is that only a portion of these organisms actually adhere to the corneal surface and are able to invade/colonise. The data presented suggest that 10^6^–10^7^ CFU/cornea is the maximum number that can be recovered from an ex vivo cornea, and this maximal amount occurs after 24 h.
6. In contrast to the bacterial infections, single-species infections with *C. albicans* and *F. solani* did not show a significant increase in the recovery of organisms after 48 h versus 24 h, and the numbers of organisms recovered were lower, despite the same inoculum. This has been described briefly in the literature, where as many as 10^9^–10^6^ CFU/cornea for *C. albicans* and 10^3^ CFU/cornea for *F. solani* were inoculated into in vivo or ex vivo murine, rabbit or rat models, with recovery of as little as 10^5^–10^3^ *C. albicans* CFU/cornea [12, 44–46], and 10^3^ *F. solani* CFU/cornea [47], respectively. The reason for this is not fully understood.
7. From the 24-h histology images presented here, little infiltration of fungi into the corneal tissue is seen, with organisms remaining predominantly at the surface. However, particularly for *F. solani*, there was vast infiltration of fungal cells throughout the stroma after 48 h. The histology of infected in vivo cultures mimics histological images of clinical infection, with a dense white fungal plaque, corneal opacity, corneal infiltration, oedema, ulcer formation, satellite lesions, corneal neovascularisation and hypopyon [47–51]. Yeast forms of *C. albicans* and conidia of *F. solani* are shown to adhere to the stroma, and after a period of time, hyphae form that penetrate the stroma [12, 45, 52] to a depth of approximately 150 μm [44]. This was observed in our ex vivo rabbit and human cornea models.
8. Differences were observed between rabbit and human corneas primarily in the number of organisms recovered from each cornea, i.e. there was an approximately tenfold increase in the recovery of bacteria from human versus rabbit corneas. This may have been due to the use of different bacterial strains (ATTC strains [human] and local clinical strains [rabbit]), intrinsic differences between the corneas of the two species, including anatomical and molecular differences such as differences in the Bowman’s membrane [44] arrangement of collagen fibres [53], size, thickness [54], secretion of antimicrobial peptides [55], or surface mucin modifications [56]. Furthermore, the difference in bacterial recovery could be due to the use of stationary-phase organisms in rabbit experiments and log-phase bacteria in human corneal experiments. However, in comparing these two types of inocula, we have established that a similar number of bacteria in either phase still results in a clinically relevant level of infection for both single- and multi-species infection in both corneas, showing comparable histology.
9. The length of time these models were cultured in vitro was short. The acute nature of such an infection limits its use to short-term experiments involving, for example, investigation of treatment strategies [57], innate immune responses [58], detection of organisms [59] and host–microbe interactions [18]. These models were not intended to replicate the clinical outcome of infection that can be observed in vivo, which may develop over several weeks when not treated effectively. In these ex vivo models, there is an obvious lack of a host immune component, and the presence and infiltration of inflammatory cells is thought to contribute to the severity of disease [26]. As such, these ex vivo models do not form corneal ulcers as typically observed clinically [18, 41, 48]. They also lack tear films, which play a defensive role.

In summary, we achieved our aim of establishing a reproducible infection of both human and animal corneas. It is certain that no model system (including animals) is a perfect surrogate for the natural human infection. Nonetheless, useful data can still be obtained. We show that we can establish a reproducible in vitro bacterial and fungal infection, with the final number of recoverable bacteria/fungi comparable to that from natural in vivo experiments. These models are now being used in the evaluation of microbial detection systems.
